# Evaluation of Genetic Polymorphisms of *N-acetyltransferase 2* and Relation with Chronic Myeloid Leukemia

**DOI:** 10.31557/APJCP.2020.21.12.3711

**Published:** 2020-12

**Authors:** Entesar M Tebien, Hiba B Khalil, Jeremy Mills, Abozer Y. Elderdery

**Affiliations:** 1 *Faculty of Medical Laboratory Sciences, Department oF Hematology, Al Neelain University, Sudan. *; 2 *Faculty of Applied Medical Sciences, Clinical Laboratory Sciences, Shaqra University, Saudi Arabia. *; 3 *Faculty of Medical Laboratory Sciences, Al Neelain Stem Cell Center, Al Neelain University, Sudan. *; 4 *School of Pharmacy and Biomedical Sciences, University of Portsmouth, UK. *; 5 *Faculty of Applied Medical Sciences, Clinical Laboratory Sciences, Jouf University, Saudi Arabia. *; 6 *Faculty of Medical Laboratory Sciences, Department of Hematology, University of El Imam El Mahdi, Sudan. *

**Keywords:** CML. NAT2, single nucleotide polymorphism

## Abstract

**Objectives:**

The *N-Acetyltransferase 2 (NAT2)* gene encodes a key enzyme involved in xenobiotic metabolism, which contributes to the detoxification of numerous cancer therapy-induced products. However, the NAT2 genotype/phenotype is not fully understood and few studies have reported its relationship with CML. The aim of this study was to determine whether its polymorphisms (*C481T, G590A, 803A>G* and *857G>A*) have a role in chronic myeloid leukemia susceptibility (CML) in Sudanese population.

**Methods:**

We performed a case- control study. DNA from 200 CML patients and 100 controls was analyzed for the *NAT2* polymorphisms using PCR-RFLP assay.

**Results:**

The study showed *NAT2 *polymorphisms 803AG are associated with CML protection by a factor of 2.3, (OR = 0.044, 95% CI: 0.020-0.095, p = 0. 000). The study indicated that the heterozygous (GA) and mutant (AA) variants of the G857A genotype also offer protection, (OR = 0.002, 95% CI: 0.002-0.019, p = 0. 000) and (OR = 0.018, 95% CI: 0.002-0.133, p = 0. 000), respectively.

**Conclusion:**

There was no significant difference in CML diagnosis among Sudanese cases with the 481C→T and 590G→A polymorphisms. But patients with the compound NAT2 genotypes 481CT/803 AG, 590AG/ 803AG, 590AG/ 803GG, 590AA/ 803AG and 590GG/ 803AG were found to have a reduced risk. The current study demonstrates that polymorphisms of NAT2 A803G and G857A might also act as protective factors against developing the disease.

## Introduction

Chronic myeloid leukemia (CML) is one of a group of diseases referred to as a myeloproliferative disorder of pluripotential hemopoietic stem cells (HSCs). Here, chromosomal translocation leads to an oncogenic *BCR–ABL* gene fusion, which enhances tyrosine kinase (TK) activity. Anomalously activated kinases disturb downstream signaling pathways and cause abnormal proliferation, differentiation and resistance to cell death (Flis and Chojnacki, 2019). Cancer susceptibility and CML is related to inherited differences in the capability of xenobiotic enzymes to eliminate foreign agents (Kassogue et al., 2015). xenobiotic metabolizing enzymes are a primary line of defense against bioactive invaders present in tissues and organs (Rekka et al., 2019).

Xenobiotic metabolizing enzymes are classified into Phase I enzymes: (Cytochrome p450), Phase II enzymes (Glutathione-s-transferases, N-acetyltransferases), and Phase III systems, comprising multidrug resistance-associated proteins (MRPs) (Wu and Lin, 2019; Arana et al., 2016). Acetylation is a critical processes of xenobiotic metabolism and involves catalysis by acetyl CoA-dependent aryl amine N-acetyltransferase (NAT) enzymes. This plays an essential role in protein synthesis, drugs biotransformation and DNA regulation (Santos et al., 2016). 

NAT2 is located on chromosome 8 (8p21.3-23.1 and 8p22) (Hernández-González et al., 2018) and is a Phase II drug-metabolizing enzyme. It catalyzes the detoxification of common carcinogens and the acetylation of numerous clinically used drugs eg: isoniazid, sulfonamides, procainamide, and hydrazine (Adithan and Subathra, 2016). 

The *NAT2* gene is highly polymorphic with more than a hundred described variant alleles (Aklillu et al., 2018), and alterations in its structure lead to a variety of responses such as susceptibility of cancer and adverse drug reactions (Dursun et al., 2018). Its polymorphisms are classified as rapid, intermediate, and slow acetylation phenotypes, dependent on the degree of alteration of gene and protein products (Salazar-González et al., 2018; Selinski et al., 2015). 

Several studies have attempted to explain the association between NAT2 polymorphism and numerous cancer types including breast, lung cancer, acute lymphoblastic leukemia and acute myeloid leukemia (Liu et al., 2015; Zou et al., 2017; Hara et al., 2017). However, there are only a limited number of published studies on its association with CML (Ouerhani et al., 2011; Lemos et al., 1999), and this study focuses on the impact of NAT2 (C481T (rs1799929), G590A (rs1799930), A803G (rs1208) and G857A (rs1799931) polymorphisms on the disease (Santos et al., 2016).

## Materials and Methods

The study comprised 200 patients with CML (Philadelphia chromosome positive), and 100 healthy control subjects. Transcript analysis was performed in the cytogenetic lab in the Radiation and Isotopes Center of Khartoum (RICK), between August 2014 and August 2017. Subject consent was obtained from donors and the protocol ratified by the research committee of the Faculty of Medical Laboratory Science at Al-Neelain University.

CML diagnosis was based on hematological features, cytogenetics and the detection of BCR-ABL. 

*Genotyping analysis*

A sample of EDTA anticoagulated blood (3ml) was collected from each subject for hematological and molecular analysis. A blood cell count was first performed with an automated cell counter (Sysmex KX-21N) and the DNA then extracted by the guanidine chloride procedure. Genotyping of NAT2 was achieved by polymerase chain reaction and restriction fragment length polymorphism (PCR-RFLP) (Michael et al., 2018).

PCR amplification was undertaken with published primers (Oqal et al., 2012), and the polymorphic reaction performed using a Protothermal Biometra T Advanced Cycler - under the protocol designated by computer program; Optimase Protocol Writer™(touch down PCR. 

RFLP analysis was performed to manufacturer’ instructions using endonuclease KpnI, DdeI, TaqI, and BamHI for the detection of mutations C481T, C590A, A803G and G857A, (NEW ENGLAND Bio Labs), respectively (Ben et al., 2017).

Post PCR, the fragment length for all NAT2 polymorphisms were 547bp and previously reported restricted enzymes under PCR-RFLP analysis is listed in Table 1.

*Statistical analysis*

The Normality test was used for quantitative data with non-parametric data analyzed using ANOVA and Kruskal Wallis. A Chi square test was also used within and between groups and logistic regression was utilized to calculate Odds ratios (ORs). 95% confidence intervals (CI) were calculated using SPSS 25.0 software (SPSS Inc, Chicago, IL, USA), and a p-value benchmark of < 0.05 was considered statistically significant.

## Results

The frequency of NAT2 polymorphisms according to gender among the patients was displayed in Table 2. A total of 200 CML patients were enrolled in this study. CML patients comprised of 132 males and 68 females. Regrading to *NAT2* gene C481T polymorphism in the case group, the frequencies of the males were carrying CC, CT, and TT genotypes were 1.5%, 12.5%, and 52%, with a frequency of 0.5%, 6%, and 27.5 in the females. Whereas the frequencies of genotypes AA, AG, and GG in* NAT2* gene A803G polymorphism in the male were 36.5%, 9%, and 20.5%, and 19%, 6.5% and 8.5% in females, respectively. The GG, GA, and AA genotypes of *NAT2 G590A* polymorphism were 14%, 38.5%, and 13.5% among the males, and 5.5%, 20.5%, and 8% in the females. Finally, the males carrying the GG, GA, and AA genotypes of *NAT2* gene G857A SNP were 23.5%, 0.5%, and 42%, whereas, in the females, 15.5%, 0.5%, and 18% respectively. None of the differences between genders were statistically significant at (P>0.05). Therefore, CML patients showed no statistical difference in NAT2 genotyping according to gender.

The distribution by age with NAT2 genotypes among case groups with CML, is shown in Table 3. The overall mean age of CML patients was 45.06 ±12.337yrs. The mean age of individuals carrying CC, CT, TT genotypes of *NAT2 C481T* gene polymorphism were 49.5 ±5.6, 45.41 ±11.8, and 44.86 ±12.7yrs, respectively. The mean age of CML patients carrying AA, AG, and GG of NAT2*5G (A803G) gene polymorphism were 46.12 ±11.8, 43.06 ±10.5, and 44.09 ±14.2yrs, respectively. In the case of *NAT2 (G590A)* gene polymorphism, the mean age of the individuals carrying the GG, GA, and AA genotypes were 44.79 ±12.7, 45.23 ±12.3, and 44.81 ±12.4yrs respectively. Whereas, the mean ages of individuals carrying GG, GA, and AA genotypes were 45.69 ±11.1, 50.00 ±11.3, and 44.56 ±13.2yrs respectively. The data showed that no significant differences were observed with age regarding the presence of NAT2 C481T, A803G, G90A ,and G857A genotypes.

The distribution of NAT2 C481T, A803G, G590A and G857A genotypes and alleles in CML patients and the control was also taken, with the following results:

Frequency of the NAT2481 CC genotype was slightly higher among CML patients (2.0%) when compared to the control (1.0%). Additionally, 481CT genotype frequency was slightly higher among CML patients (18.5%) than the control (15.0%), but not of statistical significance (p = 0.677). 

A similar statistical difference was found in frequency of the 481 TT genotype, which was only slightly higher in the control (84.0%) than the patient group (79.5%), (OR = 0.473, 95% CI:0.052-4.302, p = 0. 506). Moreover, little difference in T allele frequency was found, it being recorded in 92.5% of the control and 88.75% of CML patients, with a p =0.149. 

From NAT2 A803G samples however, frequency of 803AA was significantly higher among CML patients compared to the control, its presence being 55.5% and 29.0%, respectively (OR =0.044, 95% CI:0.020-0.095, p = 0. 000).

Frequency of the homozygous mutant 803GG and heterozygous 803AG was higher in the control than CML patients. Incidence of the homozygous mutant 803GG was found in 26% of the control as opposed to 10% of CML patients, (OR = 0.201, 95% CI:0.091-0.445, p = 0. 0.000), and presence of the heterozygous 803AG was 64% in the control and just 15.5% of CML cases (OR = 0.44, 95% CI: 0.020-095, p = 0. 000).

The mutant allele G was also significantly higher in control subjects when compared with cases at 58% and 36%, respectively.

From NAT2 G590A samples, 590GG and 590AA genotype distribution was higher among CML patients (29.5%) and (11.5%) respectively, compared to control group (23.0%) and (10.0%). With OR = 0.897, 95% CI: 0.370-2.137 and p = 0. 809 however, this difference was not considered significant. 

NAT2 590AG was higher in the healthy control (67%) than CML patients (59%), but again, with OR = 0.687, 95% CI:0.389-1.211 and p = 0.194, this difference was not significant. Accordingly, presence of the G allele was higher in CML patients (59.0%) compared to the control (56.5%), but again of no significant difference (p = 0. 558). NAT2 857GG was also higher in patients (39.5%) compared to the control (1.0%).

Frequency of 857GA and 857AA in the NAT2G857A genotype was higher in the control at 16.0% and 83.0% respectively, as opposed to 1.0% and 59.5% of CML patients. This difference was highly significant, (OR = 0.002, 95% CI:0. 000-0.19, p = 0. 0.000) and (OR = 0.018, 95% CI:0.002- 0.133, p = 0. 0.000) respectively. 

Mutant allele A presence was significantly higher in the control, it being found in 92% of healthy participants as opposed to 60% of CML cases, (p = 0. 0.000). Furthermore, logistical analysis of carriers of the homozygous mutant heterozygous to 1208 and rs1799931, were both associated with CML protection - unlike those with the homozygous wild type allele, (OR = 0.89, 95% CI: 0.044 -0.181, p=0.000) and (OR = 0.015, 95% CI: 0.002-0.0113, p=0.000), respectively, Please see results in Table 4.

Frequency of NAT2 genotype combinations among patients and the control is found in Table 5. Within the NAT2C481T and NAT2GA803G combination, CTAGwas the only genotype represented significantly more in patients (11%) compared to control (0.5%) with (OR = .023, 95% CI: (.001 -.456, p = .013). CTAG is therefore associated with protection from CML.

Additionally, five polymorphisms were considered highly significant in decreasing CML risk and these had a synergistic effect on each other. They were represented in the following combinations:

NAT2481CT/NAT2 803AG (OR = 0.023, 95% CI: 0.001 -0.456, p = 0 .013)

NAT2 590GG/ NAT2803AG (OR = 0.006, 95% CI: 0.001 -.057, p = 0 .000)

 NAT2 590AG/ NAT2 803AG (OR = 0.076, 95% CI: 0.21 -0.274, p= 0.000)

 NAT2590AG/ NAT2803GG (OR = 0.121, 95% CI: 0.032 -0.448, p = 0.000)

 NAT2 590AA/ NAT2803AG (OR = 0.038, 95% CI: 0.007 -0.197, p = 0.000) 

NAT2 590GG/ NAT2803AA (OR = 0.006, 95% CI: 0.001 -0.057, p = 0.000).

**Table 1:** Restriction Fragment Pattern of Various NAT2 Polymorphisms Enzyme Units BP Fragments

Polymorphism	Enzyme -unite	Wild type	Mutant type
481C>T	Kpn 1(2U)	CC: 433 and 114 bp	Heterozygous CT: 547,433 and 114bp
			Homozygous TT: 547bp
857G>A		GG:490+57 bp	Heterozygous GA: 547,490 and 57bp
			Homozygous AA: 547bp
590G>A	BamH 1(4U))	GG:222,170 and155bp	Heterozygous GA:392,222,170 and 155 bp
			Homozygous AA:392 and 155 bp
803A>G	Taq1(4U)	AA: 345 ,137and 65 bP	Heterozygous AG: 345,137,114 and 65bp
			Homozygous GG:345,114and 65 bp

**Table 2:** Frequency of NAT2 Genotyping According to Gender among Patient

Genotyping		Male	Female	P-value
NAT2*5A (C481T)	CC	3 (1.5%)	1(0.5%)	0.9
	CT	25 (12.5%)	12 (6%)	
	TT	104 (52%)	55 (27.5%)	
NAT2*5G (A803G)	AA	73 (36.5%)	38 (19%)	0.487
	AG	18 (9%)	13 (6.5%	
	GG	41 (20.5%)	17 (8.5%)	
NAT2*6B (G590A)	GG	28 (14%)	11 (5.5%)	0.667
	GA	77 (38.5%)	41 (20.5%)	
	AA	27 (13.5%)	16 (8%)	
NAT2*7B (G857A)	GG	47 (23.5%)	31 (15.5%)	0.328
	GA	1 (0.5)	1 (0.5)	
	AA	84 (42%)	36 (18%)	
Total		132 (66%)	68 (34%)	

**Table 3:** Age Distribution According to NAT2 Genotypes among Case Group with CML

Genotype (N)		Mean ±SD	P- value
NAT2*5A (C481T)	CC (4)	49.5 ±5.6	0.608
	CT (37)	45.41 ±11.8	
	TT (159)	44.86 ±12.7	
NAT2*5G (A803G)	AA (111)	46.12 ±11.8	2.15
	AG (31)	43.06 ±10.5	
	GG (58)	44.09 ±14.2	
NAT2*6B (G590A)	GG (39)	44.79 +12.7	0.83
	GA (118)	45.23 ±12.3	
	AA (43)	44.81 ±12.4	
NAT2*7B (G857A)	GG (78)	45.69 ±11.1	0.658
	GA (2)	50.00+11.3	
	AA (120)	44.56+ 13.2	
Mean age		45.06 ±12.337	

**Table 4:** Association between NAT 2 C481T, A803G, G590A and NAT2 G857Agenotype and Risk of CML

Polymorphisms		Patient N = 200 (%)	Control N= 100 (%)	Odd ratio	95% CI	P-value
NAT2C481T	CC	4 (2.0)	1 (1.0)	Ref		
	CT	37 (18.5)	15 (15)	0.617	0.064-5.981	0.677
	TT	159 (79.5)	84 (84)	0.473	0.052-4.302	0.506
	C allele	45 (11.3)	15 (7.5)	0.639	0.85-2.88	0.149
	T allele	355 (88.8)	185 (92.5)			
NAT2A803G	AA	111 (55.5)	10 (10.0)	Ref		
	AG	31 (15.5)	64 (64.0)	0.044	0.020-.095	0.000*
	GG	58 (29.0)	26 (26.0)	0.201	0.091-.445	0.000*
	A allele	253 (63.3)	84 (42)	0.419	1.68-3.66	0.000*
	G allele	147(36.8)	116 (58)			
NAT2 (G590A)	GG	59 (29.5)	23 (23)	Ref		
	AG	118 (59)	67 (67)	0.687	0.389-1.211	0.194
	AA	23 (11.5)	10 (10)	0.897	0.370-2.173	0.809
	G allele	236 (59)	113 (56.5)	0.793	0.64-1.27	0.0.588
	A allele	164 (41)	87 (43.5)			
NAT2 (G857A)	GG	79 (39.5)	1 (1.0)	Ref		
	GA	2 (1.0)	16 (16.0)	0.002	0.000-.019	0.000*
	AA	119 (59.5)	83 (83.0)	0.018	0.002-.133	0.000*
	G allele	160 (40)	16 (8)	0.13	4.43-0.13.7	0.000*
	A allele	240 (60)	184 (92)			

Key: Ref: reference.

**Figure 1:**
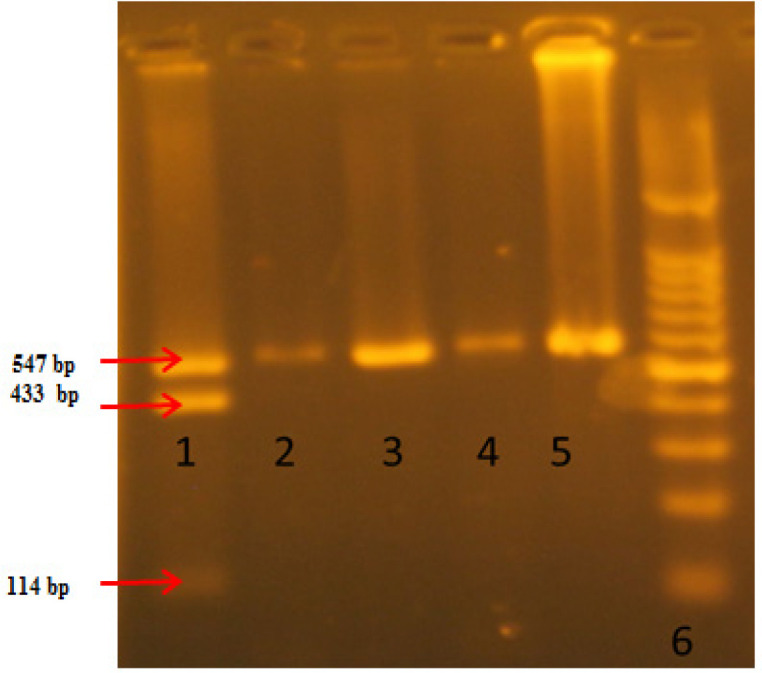
Detection of NAT2 Polymorphisms-C 481T on 4% Agarose Gel. DNA product digested with restriction enzyme KpnI for C481T,. Lane 1: heterozygous CT (547,433 and 114 pb), lane 2, 3,4 and 5( un cut product 547bp) homozygous mutant TT. Lane 6: : Molecular weight marker 100 bp

**Figure 2:**
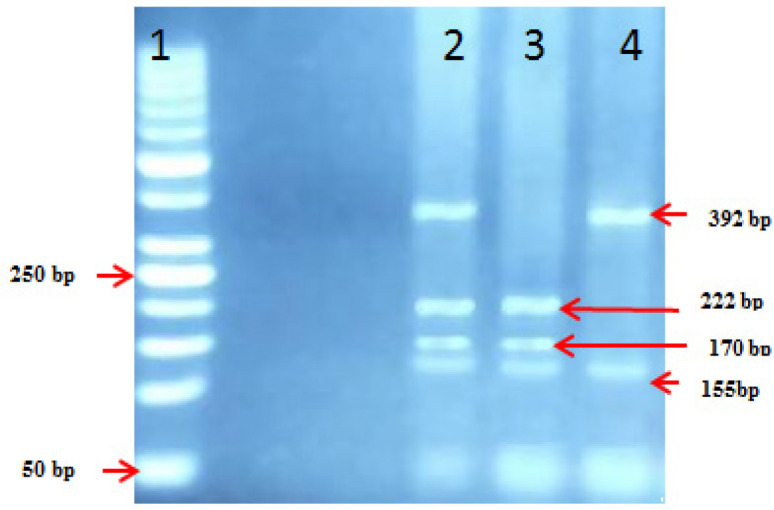
Detection of NAT2- Polymorphisms on 4% Agarose Gel. DNA product digested with restriction enzyme Taq1 for G590A, Lane 1: Molecular weight marker 50 bp. Lane 2: hetrozygous GA (392,22,170&155bp). Lane 3: wild type GG(222,170&155bp). Lane 4: homozygoyous mutant AA (392&155bp)

**Table 5:** *NAT2* Genotype Interaction Analysis in CML Patient and Control

NAT2C481T and NAT2GA803G		Case		Odd ratio	95%CI	P-value
		Control N (%)	Patient N (%)			
Comb1	CC / AA	1 (1%)	4 (2%)	Ref		
	CC / GG	1 (1%)	1 (0.5%)	0.25	(0.01- 8.56)	0.442
	CT/ AA	3 (3%)	22 (11%)	1.83	(0.15 - 22.36)	0.635
	CT / AG	11 (11%)	1 (0.5%)	0.023	(0.001 -.456)	0.013*
	CT / GG	1 (1%)	15 (7.5%)	3.75	(0.190 - 74.06)	0.385
	TT / AA	7 (7%)	84 (42%)	3	(0.294 -30.62)	0.354
	TT/ AG	52 (52%)	31 (15.5%)	0.15	(0.016 -1.39)	0.095
	TT / GG	24 (24%)	42 (21%)	0.44	(0.046 -4.14)	0.471
NAT2G590A and NAT2A803G		Control N (%)	Patient N (%)	Odd ratio	95%CI	P-value
Comb2	GG / AA	3 (3%)	32 (16%)	Ref		
	GG / AG	17 (17%)	1 (0.5%)	0.01	(0.001 - 0.057)	0.000*
	GG / GG	3 (3%)	27 (13.5%)	0.84	(0.157 - 4.53)	0.843
	AG / AA	7 (7%)	59 (29.5%)	0.79	(0.191 -3.27)	0.745
	AG / AG	38 (38%)	31 (15.5%)	0.08	(0.021-0.27)	0.000*
	AG / GG	21 (21%)	27 (13.5%)	0.12	(0.032 -.45)	0.002*
	AA / AA	1 (1%)	19 (9.5%)	1.7	(0.173 -18.37)	0.628
	AA/ AG	10 (10%)	4 (2%)	0.04	(0.007-0.19)	0.000*
	GG/ AA	3 (3%)	32 (16%)	0.141	(0.001 -0.06)	0.073*

Key: (*), denote; *, significant p. value

## Discussion

The NAT2 acetylated genotype has a large influence on cancer predisposition (Jiang et al., 2019) and may induce susceptibility to tumors arising from industrial and environmental aryl amines (Mitchell, 2019). To date, a limited number of studies have been published in Sudan concerning inter-individual and inter-ethnic differences in NAT2 allele frequencies, (Ali et al., 2019; Al-Yahyaee et al., 2007) but this is the first investigation into its genetic variations (481 C→T, 590 G→A, 803 A→G and 857G→A) and their association with CML susceptibility and progression (Kotila et al., 2019).

Our results revealed no statistical difference in the presence of the heterozygous 481CT and mutant 590AA genotypes between CML patients and the control. In patients, 481CT genotype presence was actually slightly higher in patients (18.5%) than the control (15.0%), (OR = 0.617, 95% CI: 0.064-5.981, p = 0.677). Conversely, a slightly higher 481 TT genotype frequency was found in the control (84.0%), as opposed to (79.5%) of patients group. Again however, this was not significant difference (OR = 0.473, 95% CI:0.052-4.302, p = 0.506). 

Moreover, NAT2 590GA and AA presence was of no significant difference between diagnosed CML patients and the control (OR = 0.687, 95% CI: 0.389-2.1.211, p = 0. 194) and (OR = 0.897, 95% CI: 0.370-2.173, P = 0. 809). These results are partially in agreement with Silveira and coworkers regarding NAT2 variant polymorphism presence, in ALL patients and their control (Silveira et al., 2012). The current study is also partially consistent with an earlier study conducted by Lemos and co-workers, who failed to find an association between these polymorphisms and other types of hematological malignancy(Lemos et al., 1999). Research by Ouerhani et al., (2011) investigated the association between xenobiotic metabolizing gene polymorphisms and susceptibility to *AML, CML, CLL*, and *ALL* in the Tunisian population, and found that NAT2 polymorphisms do not influence either predisposition or prognosis in CML patients. 

More specifically, studies amongst Han Chinese populations found no significant association between rs1799930 and AML susceptibility which partially concords with Dursun et al, who revealed that rs1799929, rs1799930, and rs1799931 polymorphisms of the *NAT-2 *gene are not risk factors for the development of psoriasis (Dursun et al., 2018; Zou et al., 2017). 

In contrast with our findings, another study has demonstrated that AML risk increases in patients with rapid or intermediate NAT2 genotypes (Ouerhani et al., 2011), and Gra et al showed that NAT2 genotypes 341T/T, 481C/C, 590G/G are more frequent in children with acute leukemia than in the population control (Gra et al., 2008). 

The association of NAT2 polymorphisms with the predisposition of acute leukemia has produced controversial results (Zhu et al., 2019). Gra et al., (2008) found ALL and AML patients expressed a rapid acetylation phenotype, suggesting that the increased frequency of malignancy (acute leukemia) was due to enhanced activation of aryl amines as potential pro-carcinogens. It is already been shown that the rapid acetylation phenotype occurs more frequently in patients with colon and colorectal cancer (Kabir and Rehman, 2018).

Gong et al., (2001) in addition to a meta-analysis of prostate cancer, disagree with studies undertaken on solid tumors in adults which demonstrate that NAT2 slow acetylator individuals are at a greater risk of prostate and lung cancer (Koda et al., 2017; Liu et al., 2015).

Our data demonstrates the protective effect of NAT2A 803G and G857A against CML. 803AG in its heterozygous form offers approximately 2.3 fold protection and the heterozygous (GA) and mutant (AA) in the 857G→A genotype, both decreased risk also. Our study is in harmony with Zou et al., (2017) who revealed that the *rs1799931 G>A NAT2* gene replaced glycine by glutamic acid in the 286th amino acid of the protein. The locus mutation alters enzyme activity and the further effects of drug metabolism in the activation of carcinogens, in raising or lowering cancer incidence.

A major meta-analysis has reported rs1799931 as a protective factor against cancer progression (Tian et al., 2014). However, Kamel et al., (2015) reported that NAT2*5B (314T>C, 481C>T and 803A>G) & *5C (314T>C,803A>G) were associated with a decreased risk of ALL development, giving 1.8 and 2.4-fold protection, respectively.

This study agrees with our finding that the 803A>G polymorphism is associated with CML protection, to some extent. Furthermore, we found that risk also decreased as a result of the *G590A* gene polymorphism, in its significant relationship with A803G and C481T - when in interaction with A803G. CML patients with compound *NAT2 590GG/ NAT2803AG, NAT2 590AG/ NAT2803AG, NAT2 590AG/ NAT2803GG, NAT2 590AA/ NAT2803AG* and NAT2 *590GG/ NAT2803AA* genotypes were found to have a decreased risk.

The present results reveal these combinations as having a protective effect against CML development. However, Gra et al., (2008) found that the combination of GST and NAT2 genotypes greatly increased the risk of acute leukemia in childhood. This risk frequency was higher than those with the GSTT1 null genotype, GSTM1 null genotype, or NAT2 genotypes; 341T/T, 481C/C and 590G/G.

A previous study undertaken by Zheng et al., (2017) also documented the significant risk of ALL with carriers of rs1799931 and rs1801280, and that carriers of the rs1799931-GA/AA and rs1801280-TC/CC genotypes had the highest risk of all. 

Our study has several limitations specifically that more SNPs within the *NAT2* gene should have been studied and sample size should have been larger. In addition, the CML phases should receive greater focus.

In conclusion, there is evidence that both NAT2A 803G and G857A polymorphisms were associated with protection from CML in the Sudanese population. Also, the gene-gene interaction between *NAT2 590GG/ NAT2803AG, NAT2 590AG/ NAT2803AG, NAT2 590AG/ NAT2803GG, NAT2 590AA/ NAT2803AG* and *NAT2 590GG/ NAT2803AA* genotypes were associated with a decreased CML risk. However, there was no such association between C481T and G590A and CML.

## Data Availability

The data that support the findings of this study are available from the corresponding author, [AE], upon reasonable request.
